# Yeast Sen1 Helicase Protects the Genome from Transcription-Associated Instability

**DOI:** 10.1016/j.molcel.2010.12.007

**Published:** 2011-01-07

**Authors:** Hannah E. Mischo, Belén Gómez-González, Pawel Grzechnik, Ana G. Rondón, Wu Wei, Lars Steinmetz, Andrés Aguilera, Nick J. Proudfoot

**Affiliations:** 1 Sir William Dunn School of Pathology, University of Oxford, South Parks Road, Oxford OX1 3RE, UK; 2 Centro Andaluz de Biologia Molecular y Medicina Regenerativa CABIMER, Universidad de Sevilla-CSIC, Avenida Americo Vespucio s/n, Sevilla 41092, Spain; 3 European Molecular Biology Laboratory, Meyerhofstrasse 1, 69117 Heidelberg, Germany

## Abstract

Sen1 of *S. cerevisiae* is a known component of the NRD complex implicated in transcription termination of nonpolyadenylated as well as some polyadenylated RNA polymerase II transcripts. We now show that Sen1 helicase possesses a wider function by restricting the occurrence of RNA:DNA hybrids that may naturally form during transcription, when nascent RNA hybridizes to DNA prior to its packaging into RNA protein complexes. These hybrids displace the nontranscribed strand and create R loop structures. Loss of Sen1 results in transient R loop accumulation and so elicits transcription-associated recombination. *SEN1* genetically interacts with DNA repair genes, suggesting that R loop resolution requires proteins involved in homologous recombination. Based on these findings, we propose that R loop formation is a frequent event during transcription and a key function of Sen1 is to prevent their accumulation and associated genome instability.

## Graphical abstract

**Graphical Abstract:**

**Figure:**
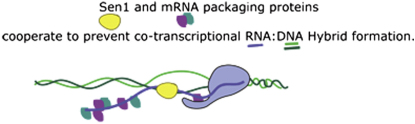

**Highlights:**

► Nascent RNA forms hybrids with underwound DNA upstream of elongating Pol II ► Single-stranded DNA so formed is prone to damage which results in genome instability ► Sen1 helicase acts to remove R loops by resolving RNA:DNA hybrids ► Sen1 function in Pol II elongation and termination may relate to R loop resolution

## Introduction

In *S. cerevisiae* nascent transcripts formed by RNA polymerase II (Pol II) on protein-coding genes are immediately processed, packaged, and exported to the cytoplasm (Luna et al., 2008; Moore and Proudfoot, 2009). Messenger RNA (mRNA) packaging protects transcripts from degradation, but also the DNA template from invasion of nascent RNA into the DNA duplex behind elongating Pol II (Aguilera and Gómez-González, 2008). The resulting RNA:DNA hybrid exposes single stranded (ss) nontemplate DNA, a structure referred to as an R loop. R loop formation has been associated with increased occurrence of transcription-associated mutation (TAM) or recombination (TAR), presumably because both induced and spontaneous lesions are more likely to occur on ssDNA. Thus, deletion of genes encoding the THO (Thp2, Hpr1, Mft1, and Tho2) and THSC or TREX-2 (Thp1, Sac3, Sus1, and Cdc31) complexes required for mRNP formation in *S. cerevisiae*—or, similarly, the splicing factor ASF/SF2 in metazoans—increase levels of R loop formation and consequently TAM and TAR (Chávez et al., 2000; Fischer et al., 2002; Gallardo and Aguilera, 2001; González-Aguilera et al., 2008; Huertas and Aguilera, 2003; Li and Manley, 2005). R loop formation in these mutants may also be connected to Pol II stalling, consequently interfering with processive elongation (Mason and Struhl, 2005; Rondón et al., 2003) and RNA processing (Libri et al., 2002; Rougemaille et al., 2008). Similarly, DNA replication may be compromised when replication forks encounter R loops or a stalled Pol II (Wellinger et al., 2006).

Although little is known about R loop resolution in yeast, in mammals their formation and resolution play a productive role in the stimulation of class switch recombination (CSR) and somatic hypermutation (SHM) in clonally expanding B cells (Yu et al., 2003). Both processes are initiated by activation induced deaminase (AID) (Muramatsu et al., 1999). Double-strand breaks (DSBs) subsequently trigger CSR via nonhomologous end joining (NHEJ) (Yu and Lieber, 2003). Although *S. cerevisiae* does not express AID, ectopically expressed AID can recognize ssDNA in R loops as a substrate when expressed in mRNA packaging mutants (Gómez-González and Aguilera, 2007; González-Aguilera et al., 2008).

Many events during transcription are orchestrated by proteins binding to the carboxy-terminal domain (CTD) of the Pol II largest subunit. CTD consists in yeast of 26 hepta-peptide repeats (YSPTSPS) that are dynamically modified during transcription. In particular, serine phosphorylation occurs during early (ser5-, 7-P) and late (ser2-P) elongation phases to allow stage specific binding of elongation and RNA processing factors (Kim et al., 2009; Komarnitsky et al., 2000). Transcription termination is also directed by different CTD-bound proteins that recognize specific sequences on the emerging nascent RNA. For protein-coding genes, this requires polyA (pA) site recognition by a ser2-P CTD bound multicomponent cleavage and polyadenylation complex (CF IA/B and CPF), as well as degradation of the downstream RNA by Rat1 exonuclease (Gross and Moore, 2001; Kim et al., 2004b; Meinhart and Cramer, 2004).

Termination of many noncoding RNAs requires an additional component, the NRD complex (Sen1, Nab3, and Nrd1), in which Nrd1 is bound to ser5-P CTD (Steinmetz et al., 2001; Vasiljeva et al., 2008). NRD-dependent termination also requires recognition of frequent short RNA sequences by Nrd1 and Nab3 (GUAA/G and UCUU respectively) (Carroll et al., 2007), although the exact sequence and NRD component requirements may vary for different terminators (Kuehner and Brow, 2008). Nrd1 ser5-P CTD specificity confines this termination pathway to transcriptional stages in which ser5-P CTD prevails and leaves promoter distal Nrd1/Nab3 binding sites unrecognized (Arigo et al., 2006a; Gudipati et al., 2008). Furthermore, as Nrd1 interacts with the exosome, NRD-terminated RNA is either degraded to protein protected stable transcripts (e.g., snoRNAs) or completely, as is the case with cryptic unstable transcripts (CUTs) (Arigo et al., 2006b; Thiebaut et al., 2006; Vasiljeva and Buratowski, 2006). Recent genome-wide transcription profiling studies reveal the wide extent of CUTs produced by Pol II and terminated by NRD. This further emphasizes the biological importance of NRD-dependent termination (Neil et al., 2009; Xu et al., 2009). Importantly, both termination pathways can substitute for each other and so provide mutual fail-safe termination mechanisms (Kim et al., 2006; Rasmussen and Culbertson, 1998). Thus, NRD termination is also important to rescue polymerases that fail to terminate at a polyA signal, especially on highly transcribed genes. Interestingly, these genes show a particular requirement for Sen1 (Rondón et al., 2009).

*SEN1* codes for a 240 kDa superfamily I helicase (DeMarini et al., 1992), and its *S. pombe* homolog possesses 3′-5′ nucleic acid unwinding activity (Kim et al., 1999). The essential C terminus contains the helicase domain, a nuclear localization sequence (NLS), and a domain necessary for interaction with the Glc7 phosphatase component of CPF (Nedea et al., 2008; Ursic et al., 1995; Winey and Culbertson, 1988). The Sen1 975 N-terminal amino acids, although dispensable for growth, interact with Pol II, RNase III endonuclease Rnt1, and the nucleotide excision repair endonuclease Rad2 (Ursic et al., 2004). Mutation of the Sen1 helicase domain results in direct and indirect pleiotropic defects in transcript processing and termination, leading to a perturbed genome-wide profile of Pol II and defective Pol I transcription termination (Kawauchi et al., 2008; Rasmussen and Culbertson, 1998; Steinmetz et al., 2001, 2006; Ursic et al., 1997). Although the severe character of these phenotypes may be explicable by the limiting presence of Sen1 in NRD (as it is only present at 125 copies/cell) (Ghaemmaghami et al., 2003), they have not been clearly attributed to a molecular function of Sen1. Employing the temperature sensitive *sen1-1* mutant (helicase domain G1747D), we set out to characterize the molecular role of Sen1 in transcription termination. We now identify broad functions for Sen1 during Pol II transcription in reducing R loop formation and consequent prevention of transcription-associated genome instability.

## Results

### Role of Sen1 Helicase Domain in Transcription Termination

**Figure 1:**
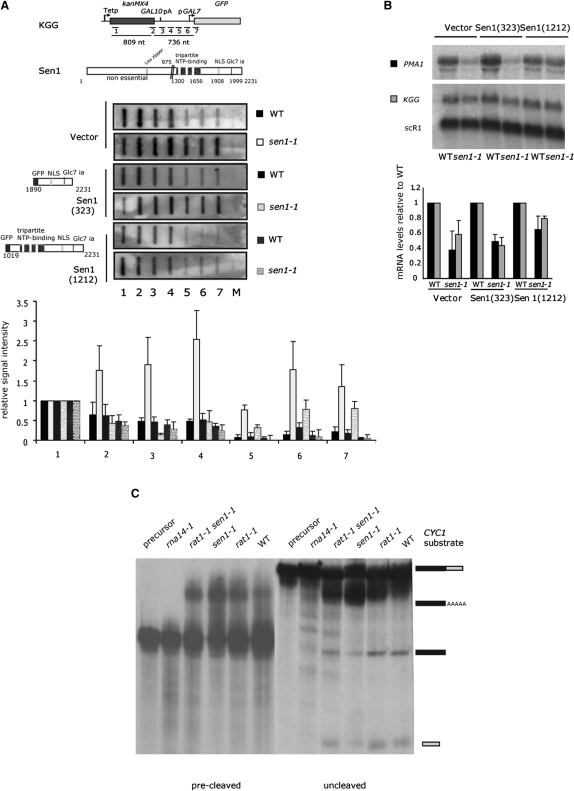
Sen1 Helicase Is Required for Transcription Termination but Not Transcript 3′ Processing (A) Top: pKGG and positions of ssM13 probes (1–7). Domain structure of Sen1. Middle: representative TRO filters of WT and *sen1-1* cells cotransformed with KGG and empty vector, Sen1(323), or Sen1(1212) constructs. Transformants were grown for 150 min at 37°C before TRO. Bottom: quantification based on four repeat experiments. (B) RNA isolated from the inocules used for TRO analysis probed for KGG and endogenous *PMA1* mRNA, as well as Pol III transcript *scR1*. Bottom: quantification of four repeat experiments. (C) In vitro cleavage and polyadenylation assays performed with extracts from WT and mutant cells grown for 150 or 90 min (*rna14-1*) at 37°C with *CYC1* 3′ pA as substrate. Positions of uncleaved, polyadenylated, cleaved, and 3′ end cleavage product are indicated. All error bars represent the standard deviation (SD). See also Figure S1.

Mutation of the Sen1 helicase domain results in genome-wide transcription termination defects of noncoding RNAs, but also of some protein coding genes (Steinmetz et al., 2006). Thus, when tested by transcription run on (TRO) experiments with the plasmid gene construct KGG (Figure 1A), with the KanMX4 gene terminated by the weak *GAL10* pA signal (Morillon et al., 2003), *sen1-1* mutants grown for 150 min at nonpermissive temperature (37°C) show a strong termination defect (Figure 1A, upper panels) (Rondón et al., 2009). This indicates either a requirement for Sen1 in Rat-dependent termination or that some transcripts over the weak *GAL10* pA signal are terminated by the NRD failsafe termination mechanism.

To determine whether Sen1 protein-protein interactions or its helicase function are required for transcription termination, we repeated TRO analysis in WT and *sen1-1* cells transformed with additional Sen1 expression constructs. Transcribed from an *ACT1* promoter, these either contained the NLS and the Glc7 interaction domain [Sen1(323)] or additionally the C-terminal helicase domain [Sen1(1212)] (Nedea et al., 2008). As shown in Figure 1A, Sen1(1212) but not Sen1(323) rescued the *sen1-1* termination defect, implying that the *sen1-1* termination defect is caused by loss of helicase function and not Glc7 mediated recruitment of CPF. We also examined steady-state mRNA produced from the KGG construct (Figure 1B). mRNA levels were reduced in *sen1-1* cells and partially complemented by coexpression of Sen1(1212), but not Sen1(323). Similarly, coexpression of Sen1(1212) restored wild-type levels of endogenous *PMA1* mRNA, also previously shown to display mild termination defects in *sen1-1* (Kawauchi et al., 2008).

The above results indicate that the Sen1 helicase domain is required both for efficient Pol II termination and mRNA accumulation. As these effects could be attributed to defective 3′ end processing, we employed an in vitro cleavage and polyadenylation assay using *CYC1* 3′ flanking RNA as the pA substrate (Figure 1C). *sen1-1* shows no defects in RNA 3′ end processing. Confirmation of this result is provided by reverse transcription analysis of *ACT1* pA usage, in which *sen1-1* showed WT pA selection (Figure S1 available online). In contrast, a CF IA mutant strain, *rna14-1*, showed the expected defect in both in vitro 3′ end processing and in vivo pA selection (Figure 1C and Figure S1). Finally, like *sen1-1*, the *rat1-1* termination mutant (or both combined) had no effect on mRNA 3′ end formation but stabilized the 3′ end cleavage product, indicative of loss of exonuclease “torpedo” function (Kim et al., 2004b; Luo et al., 2006). Overall, these combined analyses show that the Sen1 helicase is dispensable for 3′ transcript processing but is required to promote transcriptional termination.

### Mitotic Recombination Is Increased in *sen1-1*

**Figure 2:**
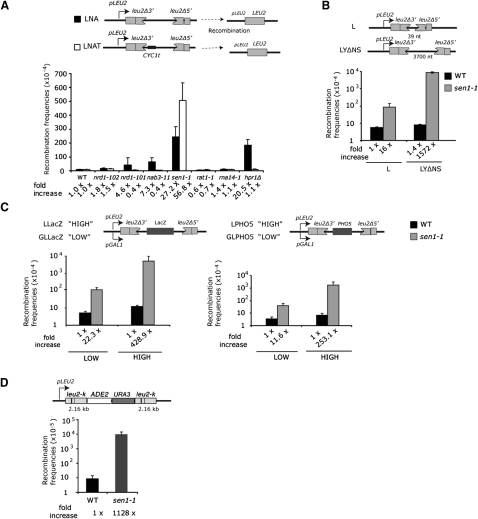
*sen1-1* Are Hyperrecombinogenic (A) Recombination substrates LNA and LNAT. Transformants were grown for 3–4 days at 30°C. Recombination generates a functional *LEU2*, allowing selection of recombinants on leu-deficient plates. Quantification of recombinants formed from six colonies of four to six transformants is presented. (B) Recombinants formed in WT and *sen1-1* transformed with L and LYΔNS containing homologous repeats separated by 39 or 3900 nt. (C) As in (B), with the LLacZ and LPHO5 substrates under control of either *LEU2* or glucose-repressed *GAL1* promoters to stimulate high or low expression. (D) Diagram and recombination quantification of chromosomal construct crossed into the WT and *sen1-1*. All error bars represent the SD. See also Figure S2.

Since *S. pombe* Sen1 can use RNA:DNA hybrids as an in vitro substrate (Kim et al., 1999), we considered the possibility that Sen1 may remove RNA:DNA hybrids formed by nascent RNA and the template strand. Such hybrids were previously shown to form in THO mutants, causing increased rates of transcription associated mitotic recombination (Huertas and Aguilera, 2003). RNA:DNA hybrids may also be naturally encountered in transcribed regions downstream of pA signals, where THO is undetectable on chromatin (Kim et al., 2004a; Luna et al., 2005). We therefore tested whether sequences downstream of a pA signal elicit TAR in *sen1-1*. We employed a plasmid borne recombination substrate that carries two truncated regions of *LEU2* overlapping by 600 nt of homologous sequence (LNA). Lack of THO elicits TAR in LNA and consequent restoration of *LEU2*, as previously shown (Figure 2A) (Prado et al., 1997). In contrast, when transcription between both repeats is terminated by insertion of the *CYC1* 38nt pA signal (*CYC1t,* LNAT), recombination levels in the *hpr1Δ* strain were reduced to background WT levels, presumably because Pol II termination restricts R loop formation. Similar analysis of LNA and LNAT transformed *rat1-1* and *rna14-1* showed no detectible increase in recombination, confirming that defects in CPF/Rat1 dependent transcription termination per se do not promote recombination (Luna et al., 2005). In marked contrast, *sen1-1* transformed with either LNA or LNAT showed high levels of recombination, suggesting that RNA:DNA hybrids may form throughout the mRNA coding region irrespective of the *CYC1t*. This lack of *CYC1t* suppression reiterates the *sen1-1 CYC1* termination defect previously reported (Kawauchi et al., 2008; Steinmetz et al., 2006). Moreover, the fact that *CYC1t* (in LNAT) further stimulated recombination may reflect an increase in R loop formation downstream of pA signals.

To determine whether this recombination phenotype was specific to Sen1, we similarly tested other NRD complex components. Although recombination levels were somewhat increased in *nab3* and *nrd1* CTD-interacting domain mutants (but not the RNA binding domain mutant *nrd1-102*) transformed with LNA, they were reduced to background levels in LNAT (Figure 2A). This suggests that these NRD mutants still recognize the *CYC1t*. Why these NRD mutations elicit some recombination is unclear at this point, but may reflect alteration in mRNP biogenesis. The fact that *CYC1t* abrogates recombination in NRD mutants but stimulates recombination in *sen1-1* clearly separates Sen1 function from Nab3 and Nrd1 and argues that Sen1 plays a distinct role outside the NRD complex.

### *sen1-1* Hyperrecombination Depends on Transcription

Hyperrecombination in THO and THSC/TREX-2 mutants shows clear transcription dependence, as it increases with greater transcript length and transcription rate but decreases when the R loop-forming RNA is removed either by RNase H activity or ribozyme directed RNA cleavage (González-Aguilera et al., 2008; Huertas and Aguilera, 2003). To test whether *sen1-1* shows a similar transcription-dependent recombination phenotype, we analyzed *sen1-1* recombination levels for various direct-repeat recombination substrates. As shown in Figures 2B and 2C, levels of recombination in *sen1-1* correlate with the length and transcriptional rate of the gene. Thus, TAR, although not abolished, was significantly decreased in two different recombination substrates when transcription from a *GAL1* promoter was glucose repressed. Finally, we verified that recombination was also stimulated between direct repeats in a chromosomal context (Figure 2D). These observations suggest that there is a correlation between transcriptional activity and *sen1-1* recombination levels.

### RNA:DNA Hybrids Form in *sen1-1*

**Figure 3:**
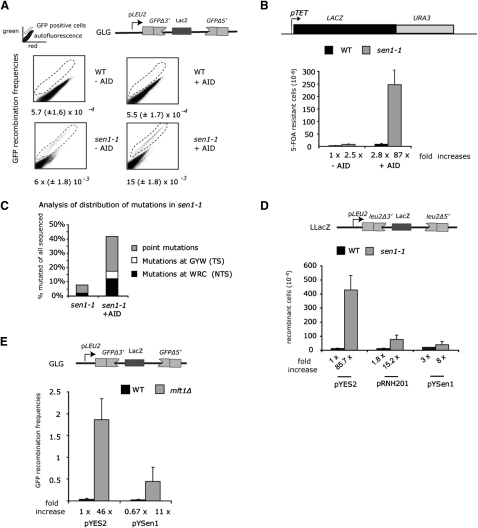
*sen1-1* Cells Form a Substrate for AID and RNase H (A) AID overexpression increases *sen1-1* TAR. pGLG recombinants forming GFP were counted after 12–16 hr growth at 30°C by FACS. AID coexpression increases GFP-positive cells in both WT and *sen1-1* strains (p = 0.018, Wilcoxon test). (B) Coexpression of AID and pLAUR-induced mutations within *URA3* in pLAUR were scored as 5-FOA resistant. (C) *URA3* sequence from mutants was amplified and sequenced. The frequency of point mutations on either strand is depicted graphically (see Figure S3 for more detail). (D) Effect of galactose-induced expression from plasmids pRNH201 (coding RNase H *RNH201*), pYsen1 (aa 1281–2231 of Sen1 cloned into pYES2), or pYES2 alone on the recombination frequencies in WT and *sen1-1* cells produced by the LLacZ system. Note that double selection and growth on galactose reduces the *sen1-1* viability and therefore recombination frequencies as compared to data in Figure 2C. (E) Recombination frequency analysis in the THO-complex mutant *mft1Δ* with the GLG recombination substrate and AID to increase recombination rates. Overexpression of Sen1 helicase reduces recombination frequency. All error bars represent the SD. See also Figure S3.

Evidence for RNA:DNA hybrid accumulation in THO mutants derives from expression of the human AID in yeast which was shown to cause a 25-fold increase in TAR (Gómez-González and Aguilera, 2007). Employing 5′ and 3′ truncated overlapping GFP repeats and intervening *LacZ* sequence as a recombination substrate, FACS analysis of GFP-positive cells showed that AID expressed in *sen1-1* also stimulates recombination albeit only 2.5-fold (P[Wilcoxon-rank-sum test] = 0.017; Figure 3A). As discussed below, this moderate but significant stimulation of TAR by AID could reflect the nature of RNA:DNA hybrids formed in *sen1-1* or be due to the fact that recombination levels in *sen1-1* cells that did not express AID were already very high.

AID C to U deamination preferentially occurs in a WRC (or GYW on the opposite strand) sequence (Pham et al., 2003). In regions of R loop formation, AID has access to the nontranscribed strand (NTS), although some mutations occur on the transcribed strand (TS) (Gómez-González and Aguilera, 2007). In an attempt to analyze the nature of AID-induced mutations in *sen1-1*, we transformed WT and *sen1-1* cells (both *ura3–*) with plasmid-encoded *LACZ::URA3* chimeric gene (pLAUR, Figure 3B) and selected AID-induced *ura3* mutations on 5-FOA (5-fluoorotic acid). Although mutation rates were very low in WT and *sen1-1* cells grown at semipermissive temperature, AID expression in *sen1-1* significantly increased mutation rates (Figure 3B). DNA sequencing of 57 5-FOA-resistant transformants revealed that 42% carried a point mutation in *URA3*, of which 71% had a point mutation within an AID sequence motif. Of these mutations 70% occurred on C (12; Fisher's test; p < 0.003) or the NTS and only 30% (five; p < 0.05) occur on G or the TS (Figure 3C and Figure S3). The small amount of WT transformants sequenced showed a distribution as earlier reported (Gómez-González and Aguilera, 2007). Since AID expression not only increased the amount of point mutations within the transcribed *URA3* in *sen1-1* but also preferentially acted on the NTS, this suggests that R loops are formed during transcription in *sen1-1* cells and displace the nontranscribed DNA strand.

We next tested whether the high recombination levels seen in *sen1-1* were sensitive to RNase H overexpression as previously observed with THO and THSC/TREX2 mutants and indicative of increased R loop formation (González-Aguilera et al., 2008; Huertas and Aguilera, 2003). Thus, RNase H overexpression (from pRNH201) reduced recombination rates of pLLacZ in *sen1-1* by 5.6-fold (Figure 3D). Furthermore, the *sen1-1* recombination phenotype correlated with deficient helicase activity and could be rescued by high copy expression of pYsen1, encoding for the Sen1 helicase domain. To exclude the possibility that increased recombination levels in *sen1-1* could be a consequence of the *sen1-1* transcription termination defect, rather than the ability of Sen1 to remove R loops, we tested the capability of Sen1 helicase domain to suppress the hyperrecombination phenotype of the THO mutant *mft1Δ*, which displays no transcription termination defect but increased levels of R loops. As shown in Figure 3E, overexpression of the Sen1 helicase domain in AID- and pGLG-transformed *mft1Δ* cells caused a substantial reduction in the number of GFP-recombinant cells. We conclude that Sen1 enzymatic activity is able to directly restrict cotranscriptionally formed R loops.

**Figure 4:**
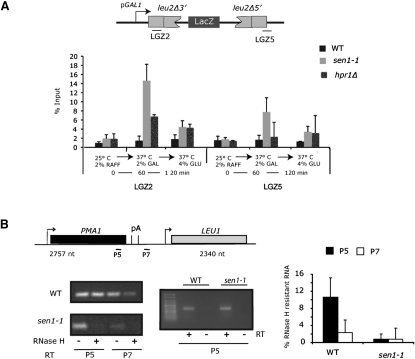
R Loops Form in *sen1-1* Cells (A) DIP analysis on pGLLacZ in *sen1-1*, *hpr1Δ*, and WT cells with antibody against RNA:DNA hybrids (S9.6). Coimmunoprecipitated DNA was detected by real-time qPCR. Inocules were grown in raffinose, induced with galactose for 1 hr at 37°C, and successively repressed at 37°C by addition of 4% glucose. (B) Reverse transcription (RT) of RNA isolated from genomic DNA preparations after treatment with RNase H. The levels of RNase H resistant RNA were measured after RT with P5 and P7 primers followed by PCR with P5 amplicon (as shown on the gene map). Signals obtained were free from DNA contamination based on minus RT controls. Left: representative gel. Right: quantification of RT normalized triplicate repeats by real-time qRT-PCR, further normalized to P5 amplicon signal obtained from non-RNase H-digested samples. All error bars represent the SD. See also Figure S4.

To obtain independent evidence for the existence of R loops formed in *sen1-1,* we employed both DNA (DIP; Figure 4A) and chromatin immunoprecipitation analysis (ChIP; Figure S4A) with the RNA:DNA hybrid-specific antibody (S9.6) (Hu et al., 2006). Yeast transformed with pGLLacZ displayed hybrid signal over the recombining *LEU2* sequence in *sen1-1* but not WT cells when shifted for 1 hr to nonpermissive temperature and induced by galactose. Hybrid signal then diminished to WT background levels when transcription was repressed by glucose addition to the medium (Figure 4A). Notably, the untranscribed origin region of pGLLacZ gave background signals (Figure S4B). Furthermore, where DIP signals were detected on LGZ2 and LGZ5 in *sen1-1* grown in galactose, these signals were sensitive to RNase H digestion prior to immunoprecipitation (Figure S4C). Overall, these data demonstrate that R loop formation is highly dynamic, closely following transcriptional activation and repression of pGLLacZ. Parallel experiments with the THO mutant strain *hpr1Δ* gave significant but lower levels of hybrid signal over the 5′ positioned *LEU* sequence (LGZ2) but not over LGZ5 (Figure 4A). The 3′ LGZ5 probe spans the *LEU2* pA signal and so will lack transcripts in *hpr1Δ* due to Pol II termination. *sen1-1* in contrast is termination defective so that read-through transcripts in this strain will still elicit R loop formation.

The S9.6 antibody was also employed in a regular ChIP analysis of endogenous *PMA1* and compared to Pol II ChIP profiles. RNA:DNA hybrid signal was detected over *PMA1* (Figure S4A), although general signal intensities were lower than those obtained by DIP analysis of the highly expressed pGLLacZ. When normalized to the gene 5′ end in WT and *sen1-1* cells, signals were detected wherever Pol II was present on chromatin (Kawauchi et al., 2008). Of note, in chromatin isolated from *rna14-1* that shows no recombination phenotype, little hybrid could be detected downstream of the pA signals, even through Pol II is still present at these positions. This may indicate that while hybrid is degraded downstream of the pA signal in presence of Sen1 and Rat1, it is stabilized in *rat1-1 sen1-1* and to a lesser extent in *sen1-1.* A modest accumulation of hybrid signal was observed over the pA signal compared to the gene body (P6 versus P5) in all tested strains and may indicate a region that is particularly prone to form RNA:DNA hybrids.

In view of the only modest RNA:DNA hybrid accumulation observed over *PMA1*, we developed an independent assay for R loop formation over this same gene (Figure 4B). Genomic DNA with associated nascent transcripts was isolated from WT and *sen1-1* cells, shifted for 1 hr to 37°C, and subjected to RNase H digestion. Subsequently, DNA was digested with DNase I and nascent RNA that survived this treatment was detected by RT-PCR with RT primers either within *PMA1* (P5) or downstream of the pA signal (P7). As shown in gel fractionation of these amplified DNAs, *sen1-1* chromatin associated RNA is highly sensitive to RNase H treatment. Quantitative PCR (qPCR) values (ratio of RNase H sensitive to total signal) show that although in *sen1-1* chromatin associated RNA is nearly fully RNase H sensitive, in WT RNase H sensitivity increases downstream of the *PMA1* pA signal. This suggests that in *sen1-1* a higher fraction of RNA forms R loop structures with genomic DNA, whereas in the WT R loops are particularly prone to forming downstream of the pA site.

From these combined analyses, we conclude that R loops accumulate in *sen1-1* in a transcription-dependent manner. Furthermore, they imply that WT-transcribed and subsequently packaged RNA still forms some level of RNA:DNA hybrids. The amount of RNA in R loop conformation appears to differ throughout the transcribed *PMA1* gene, with a particular prevalence over the pA site.

### *SEN1* Genetic Interaction with Homologous Recombination Genes

Experiments presented so far suggest that R loops formed in *sen1-1* elicit TAR. However, to substantiate this conclusion and further exclude the possibility that *sen1-1* TAR is an indirect consequence of its transcription termination defect, we employed genomic analysis. A comparison of RNA steady-state levels of *sen1-1* to WT cells grown for 150 min at 30°C was performed by hybridization to strand-specific tiling arrays (data available at http://www.ebi.ac.uk/arrayexpress/ with accession number E-TABM-863) (David et al., 2006). This revealed that among the transcripts whose expression was significantly changed (adjusted p value of < 0.01), stable unannotated transcripts were overrepresented (19% stable unannotated transcripts versus 7% for open reading frames [ORFs]), confirming a Sen1 role in the termination of some of these genes (Figure S5A and Table S4). Importantly, THO or THSC genes were absent among the 347 ORFs that were significantly changed, excluding the possibility that R loops are formed in *sen1-1* as an indirect consequence of alteration in THO or THSC/TREX-2 expression. We next considered whether the 347 significantly changed ORF transcript levels correspond to DNA damage repair and cell-cycle (DDCC) genes. DDCC genes were underrepresented, and among those changed, no clear trend was observed (Figure S5B and Table S4), with some mildly upregulated (i.e., *REC104*, *POL4*, *SMC5*, *SCM4*, and *TOP2*) and others downregulated (i.e., *DIA2*, *MMS2*, and *NEJ1*).

**Figure 5:**
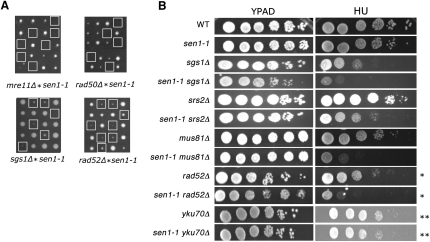
*sen1-1* Shows Synthetic Genetic Interaction with DNA Damage Repair Genes (A) Synthetic interactions between *sen1-1* and MRX gene mutations: *mre11Δ*, *rad50Δ*. Also shown are synthetic interactions between *sen1-1* and HR gene mutants *sgs1Δ* and *rad52Δ*. White boxes indicate spores that carry both mutations. (B) Analysis of HU sensitivity of double mutants grown at 25°C. Growth was compared on YPAD plates ± 50 mM HU (10 mM ^∗^ or 100 mM ^∗∗^ as indicated). See also Figure S5 and Table S4.

To define which DNA repair mechanism was induced by R loops formed in *sen1-1*, we searched for synthetic genetic interactions of *sen1-1* with mutants of candidate genes involved in either NHEJ or homologous recombination (HR). At permissive temperature (25°C) or in presence of replicative stress, we observed genetic interaction with various factors involved in HR but not in NHEJ (Figure 5). Thus, *sen1-1* (but not *nrd1-102*; Figure S5C) causes synthetic lethality with *rad50* and *mre11* deletion mutants, both found in a complex with Xrs2 and involved in initial recognition and ss resection at a DSB (Figure 5A). The critical need for HR in *sen1-1* cells is demonstrated by the phenotypes of double mutants of *sen1-1* and *rad52Δ*, *sgs1*Δ, *srs2*Δ, or *mus81Δ*. These all showed synthetic defects or displayed increased sensitivity to growth in hydroxyurea (HU). In contrast, *sen1-1 yku70Δ* double mutants showed neither growth defects nor increased sensitivity to HU, suggesting that NHEJ is not required for cell survival of *sen1-1* cells (Figure 5B). Taken together, these data suggest that proteins involved in HR but not NHEJ are important to maintain *sen1-1* viability.

### DNA Damage Foci in *sen1-1* Nuclei

**Figure 6:**
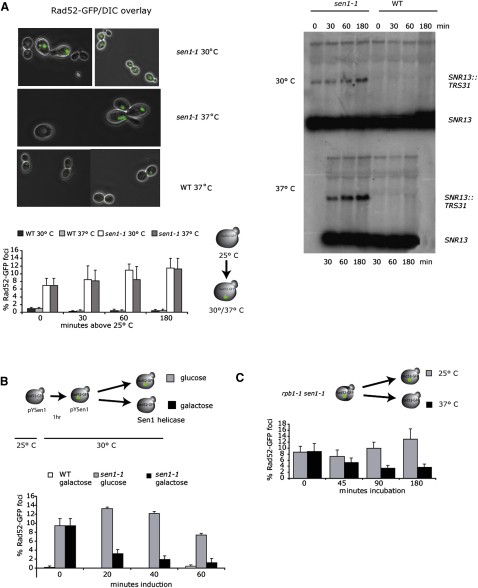
DNA Repair Foci in *sen1-1* Nuclei (A) Time course of WT and *sen1-1* grown at log phase and shifted to 30° or 37°C. At indicated time points aliquots were spotted on microscope slides and foci-containing cells scored based on 300 cells. Representative pictures (top left) and quantification of 3-5 repeats are shown (bottom left). Simultaneously isolated RNA was analyzed by Northern Blot against *SNR13* to show accumulation of bi-cistronic *SNR13-TRS31* transcript in *sen1-1.* (B) WT and *sen1-1* cells transformed with pWJ144 and pYSen1 grown in raffinose and shifted to 30°C for 1hr, before Sen1 helicase fragment expression was induced or repressed by addition of 2% galactose or 2% glucose to the medium respectively. Only WT in galactose shown and foci containing cells scored as in A. (C) *sen1-1 rpb1-1* cells transformed with pWJ144 were grown in logarithmic phase at 25°C and then shifted to 37°C. Shutoff of transcription results in decrease of Rad52 foci, either by reduced accumulation or Rad52 turnover. All error bars represent the SD.

In cells that accumulate DNA damage, factors involved in DNA repair are rapidly recruited to the damage site (Lisby et al., 2001). Consistent with the role of R loops in DNA damage as seen in *sen1-1*, we observed that many *sen1-1* cells display an accumulation of GFP-tagged Rad52 (encoded on pWJ144; Figure 6A). This percentage of *sen1-1* cells forming foci increased from 8% at 25°C to 13% when cultures were shifted to semipermissive (30°C) or nonpermissive (37°C) temperature for 3 hr. In contrast, only about 1% of WT cells formed foci under these conditions. Importantly, steady-state RNA analysis from the same cell populations showed that Rad52 foci formation correlated with accumulation of a bicistronic transcript from the *SNR13* snoRNA gene, which results from termination at the next available pA when NRD-dependent termination is defective (Rasmussen and Culbertson, 1998). As shown in Figure 6A (right panels), the *SNR13-TRS31* transcript is already present in *sen1-1* at 25°C but increases over time at 30°C and to a greater extent at 37°C. These results connect the various *sen1-1* phenotypes observed in this work and support the assumption that they are caused by mutation of Sen1 helicase domain in *sen1-1.*

Using this assay as an indicator of ongoing HR in *sen1-1*, we reinvestigated the role of Sen1 helicase in preventing or resolving transcription dependent R loops. WT and *sen1-1* transformed with Rad52-GFP and pYSen1, which encodes the Sen1 helicase domain transcribed from the *GAL1* promoter, were grown in raffinose as a neutral carbon source for 1 hr at 30°C. After addition of glucose or galactose to respectively repress or activate Sen1 helicase expression, the amount of Rad52 foci forming cells was counted. Expression of the Sen1 helicase domain rapidly reduced the number of *sen1-1* nuclei displaying Rad52 foci to almost WT levels (Figure 6B). Employing a *sen1-1 rpb1-1* double mutant, which allows rapid transcription shutdown at the nonpermissive temperature due to a mutation in the Pol II largest subunit (rpb1-1) (Ursic et al., 2004) (Figure 6C), we could also correlate the formation of Rad52 foci to transcriptional activity. When grown at 37°C, Rad52-GFP transformed *sen1-1 rpb1-1* cells displayed a time-dependent decrease in Rad52 foci as compared to growth at 25°C. Since *sen1-1* cells accumulate Rad52 foci when shifted to 37°C, these data indicate that upon transcription shutdown, Rad52 foci disappear either as DNA damage is immediately repaired or the cells die.

## Discussion

The deleterious effects of transcription on genome integrity have been suggested by various observations (Aguilera, 2002). Whenever the integrated process of transcript processing, packaging, and export in eukaryotes is disrupted, genome instability can be observed (Baaklini et al., 2004; Broccoli et al., 2004; Jimeno et al., 2002; Luna et al., 2005). This has been shown to derive from R loops, which preferentially form when mRNP biogenesis is disrupted. Our data show that Sen1 helicase plays a pivotal role in the prevention of genome instability by recombination. A large fraction of this instability is transcription dependent and linked to the formation of R loop structures. The exact nature of these structures remains to be established, but we show their accumulation can still occur with normal mRNP biogenesis. If these structures are not removed by either Sen1 helicase or RNase H directed degradation, they can exert a deleterious effect on genome stability, as is further illustrated by *SEN1* genetic interaction with HR genes. The occurrence of Rad52 foci, as a marker for ongoing recombination, shows that recombination is related to transcription, as well as to the presence of a functional Sen1 helicase domain. In summary, we suggest that R loop formation is more frequent than hitherto anticipated and requires active removal by helicases such as Sen1.

**Figure 7:**
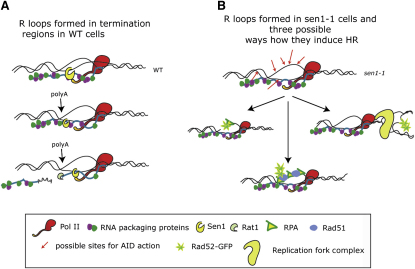
Cotranscriptional Functions of Sen1 (A) Model for Sen1 cotranscriptional function especially in termination regions. (B) Model for R loop accumulation in *sen1-1* showing three ways they may elicit HR: processing of nicks in ssDNA, ssDNA recognition, and collapse of colliding replication forks.

We suggest that as soon as the nascent transcript emerges from the polymerase body, mRNA packaging and R loop formation occur in kinetic competition (Figure 7). A fragile equilibrium between protective mRNA packaging and the hiding of specific recognition sequences is likely to exist (Bucheli and Buratowski, 2005; Bucheli et al., 2007). Therefore, RNA packaging is likely to be incomplete so leaving some transcript available for R loop formation. For pervasive CUT transcription, termination depends on NRD and by inference on Sen1 (Arigo et al., 2006b). If R loops formed in *sen1-1* extend to many CUT loci, then their accumulation, even if transient, would cover substantial regions of the genome. In both CUTs and mRNA coding genes, R loops could interfere with DNA replication, induce ssDNA breaks, or be recognized as recombination intermediates. Any of these possibilities could explain the essential need for DSB sensing proteins in *sen1-1* (Figure 5). However, the different genetic interactions of *sen1-1* and *hpr1* with HR or S phase checkpoint genes suggest structural and functional differences of the replication/recombinogenic intermediates that are formed (Gómez-González et al., 2009). Alternatively, these differences may hint at a transcription-independent role of Sen1 in DNA damage repair that is yet to be uncovered.

How may R loop accumulation in *sen1-1* be related to its transcription termination defect? R loops were originally hypothesized to slow down transcription elongation, thereby enhancing termination (Proudfoot, 1989). This would give time for the Rat1 5′-3′ exonuclease “torpedo” to catch up with Pol II but would require R loop resolution by an enzymatic activity such as Sen1 prior to degradation. Based on observations made on THO mutants, R loops have been suggested to interfere with transcription elongation (Huertas and Aguilera, 2003; Mason and Struhl, 2005). Employing *sen1-1*, in which transcript processing is normal, we predict that reduced steady-state RNA accumulation is due to reduced transcript elongation. Furthermore, the data presented here support the view that R loops preferentially form in termination regions. Thus, we employed the LNA/LNAT recombination substrates, anticipating that even though the *CYC1* pA would not elicit termination (Kawauchi et al., 2008), it should serve as a 3′ processing signal (Figure 1C), promoting disassembly of THO and consequent R loop formation (Kim et al., 2004a). Compared to LNA, *sen1-1* recombination levels increased 2-fold in LNAT. This demonstrates for *sen1-1* in contrast to THO mutants, that RNA cleavage in the context of a pA is not sufficient to relieve recombination. To reiterate this point, Figure S2 shows that in a ribozyme containing substrate, recombination levels in *sen1-1* are reduced similar to *hpr1Δ* (Huertas and Aguilera, 2003). As both ribozyme cleaved ends are unprotected they are likely to be degraded and so reduce R loop forming substrate. However, this appears not to be the case if RNA in *sen1-1* cells is cleaved at a pA, possibly as RNA downstream to the polyA signal may be less packaged and so temporarily protected from degradation by R loop formation. Although these studies require a more detailed biochemical analysis, we predict from these initial results that R loops may play a role in transcriptional termination.

In summary the molecular and genetic effects of Sen1 inactivation presented here reveal that Sen1 acts to protect the heavily transcribed genome from R loop-mediated DNA damage. Of note, mutations in the helicase domain of the human *SEN1* gene ortholog *SETX* (encoding Senataxin) cause the neurodegenerative diseases, Ataxia with Oculomotor Apraxia Type II (AOAII), and juvenile amyotrophic lateral sclerosis (ALS4). Like *sen1-1* these *SETX* mutants show defects in transcription, RNA processing, and DNA damage repair (Moreira et al., 2004; Suraweera et al., 2007, 2009). It remains to be established whether the tendency of transcription to induce R loop formation is a general feature of all eukaryotic genomes. It is possible that a range of dedicated helicases act to resolve these potentially harmful structures.

## Experimental Procedures

### Yeast Cultivation and Genetic and Cell Biology Methods

Yeast strains, plasmids, and primers are listed in Tables S1–S3. Standard methods are detailed in the Supplemental Experimental Procedures. In outline, genetic crossing of single mutants (*sen1-1* and *nrd1-102*) with HR and NHEJ mutants employed standard conditions. Recombination frequencies were scored by counting of *LEU+* cells or by FACS analysis of GFP+ cells. GFP-Rad52 nuclear foci were detected by epifluorescent microscopy.

### Transcription Run On Analysis

The transcription run on (TRO) method and probes for pKGG are as described (Morillon et al., 2003; Rondón et al., 2009).

### Northern Blot Analysis

For RNA isolation, strains were grown in minimal (selective) media at indicated temperatures. After acidic phenol RNA isolation, RNA (15 μg) was separated by 1% formaldehyde agarose gel electrophoresis. RNA immobilized on nitrocellulose membranes was detected with random primed probes.

### Chromatin and DNA Immunoprecipitation

ChIP employed real-time qPCR as previously described (Rondón et al., 2009). S9.6 purified antibody was employed for immunoprecipitation and was a kind gift from Stephen Leppla (Hu et al., 2006) and Antonin Morillon (Institut Curie, Paris). For DIP analysis, sonicated, deproteinized chromatin was immunoprecipitated with S9.6 antibody and amplified by qPCR as further detailed in the Supplemental Experimental Procedures (Liu et al., 2005). RNase H sensitivity was measured by treatment with RNase H prior to immunoprecipitation.

### RT Analysis of Genomic DNA-Associated RNA

Sequential RNase H (NEB, 2 hr at 37°C) and DNase I (Roche, 4 hr at 37°C) digestion of genomic DNA (5 μg) isolated from logarithmic phase cells cultivated for 1 hr at 37°C with yeast breaking buffer (2% [v/v] Triton X-100, 1% [w/v] SDS, 100 mM NaCl, 10 mM Tris [pH 8.0], and 1 mM EDTA [pH 8.0]), phenol, and glass beads. Isolated RNA was reverse transcribed (Invitrogen Superscript III) according to the manufacturer.

### Further Standard Experimental Procedures

These are presented in the Supplemental Experimental Procedures and include in vitro 3′ end processing, 3′end RACE, and microarray analysis.

## Supplemental Information

Document S1. Supplemental Experimental Procedures, Five Figures, and Three Tables

Table S4. Transcript Expression Levels of Three Biological Replicates, Related to Figure 5
