# Supplementary material for: Yeast Sen1 Helicase Protects the Genome from Transcription-Associated Instability
Source: Mol Cell. 2011 Jan 7;41(1):21–32. doi: 10.1016/j.molcel.2010.12.007 (PMC3314950; doi:10.1016/j.molcel.2010.12.007)
Supplement: Document S1. Supplemental Experimental Procedures, Five Figures, and Three Tables [file mmc1.pdf]

Molecular Cell, ***Volume 41***

Supplemental Information

Yeast Sen1 Helicase Protects the Genome from Transcription-Associated Instability

Hannah E. Mischo, Belén Gómez-González, Pawel Grzechnik, Ana G. Rondón, Wu Wei, Lars Steinmetz, Andrés Aguilera, and Nick J. Proudfoot

Supplemental Experimental Procedures

Yeast cultivation, genetic and cell biology methods.

Double mutant strains were generated by crossing single mutants, using standard methods. Genotypes were verified by phenotype and sequence analysis. HU sensitivity was tested by plating ten-fold serial dilutions on YPAD plates containing 10, 50 and 100 mM HU (Figure 5B). To measure recombination frequency, strains transformed with recombination substrates were grown for 3-4 days at 30˚ C. Recombination events occurring during this time were scored by comparison of total cell numbers (selected for the plasmid only) and the recombinants (selected for plasmid and the recombined *LEU2* gene). Six colonies of at least three independent transformants were analyzed. For the *sen1-1* mutation, transformants from two different spores were analyzed. Recombination substrates used are described in Supplementary Table II. GFP-based recombination was assessed in a FACScalibur of cells transformed with either pGLG alone (-AID) or co-transformed with pGLG and pGAID (+AID). After over night induction of AID and GLG in galactose, 10^6 cells were counted. AID dependent mutation was scored after counting 5-FOA resistant cells transformed with the pLAUR which encodes for *URA3* in frame to LacZ in absence or presence of pGAID. Image analysis of Rad52-GFP transformed cells was performed using epifluorescence microscopy.

DNA immunoprecipitation (DIP) analysis.

Sonicated genomic DNA was resuspended in water and 200 µg DNA diluted into 400 µl FA1 buffer (0.1 % SDS, 1 % Triton, 10 mM Hepes pH 7.5, 0.1 % sodium deoxycholate, 275mM NaCl) and immunoprecipitated with 5 µg S9.6 purified antibody for 90 minutes at 4˚ C. This was washed and eluted following regular ChIP protocols. For RNase H sensitivity experiments 200 µg DNA was incubated with 40U of RNase H (NEB) at 37°C for 2h. Reaction was stopped by adding EDTA to 10mM concentration. DNA incubated in the absence of RNase H was used as a control. Samples were diluted 10x with FA1 and DIP was performed as described above.

In vitro 3' end processing reactions.

These were performed as previously described (Kessler et al., 1996; Zhao et al., 1999), except that cells were grown in YPAD at 25˚ C and shifted to 37˚ C for 90 (*rna14-1*) or 180 (all other strains) min. The *CYC1* substrates were kindly provided by Bernhard Dichtl as described (Minvielle-Sebastia et al., 1998).

3'-end RACE

This was performed by amplifying total RNA prepared as for Northern blot analysis by reverse transcription, employing linker-coupled phased oligo dT. Specific products were amplified using a specific forward primer and the linker sequence as reverse primer. PCR products were separated on 2 % agarose gels.

RNA expression analysis by microarray. Total RNA was extracted from WT (WF1A) and *sen1-1* (WF1B) cells, grown for 150 min at 30° C in 500 ml synthetic complete medium by acid phenol chloroform extraction. 20 µg total RNA was annealed with 1.72 µg random hexamer and 0.034 µg oligodT and transcribed by Superscript RT II (Invitrogen) in presence of Actinomycin D as described in Xu et al., (2009). cDNA fragmentation, biotinylation and array hybridization was performed according to Affymetrix protocols #900671, #900720 and #900301. Micro-array hybridizations were normalized as previously described (Huber et al., 2006) and the transcript boundaries and annotations were used as published (Xu et al., 2009). Statistical assessment of differential expression between wild-type and *SEN1* mutant was done using the moderated t-test as implemented in the R-package, Limma (Smyth, 2004) and p-value adjusted for multiple testing (Benjamini and Hochberg, 1995). DDCC GO annotation was originally retrieved from www.mips.gsf.de and manually curated (van Attikum et al., 2004)

Microarray bioinformatics.

All microarray data are accessible at Array Express (http://www.ebi.ac.uk/arrayexpress). The array design is available under A-AFFY-116. We employed the genomic DNA hybridizations of Mancer et al. (2008) (E-TABM-470): recombination_060501_YJM789, recombination_060502_YJM789,recombination_060503_YJM789,recombination_060504_YJM789. cDNA hybridizations are available under the accession number E-TABM-863 (Transcription profiling of yeast *SEN1*).

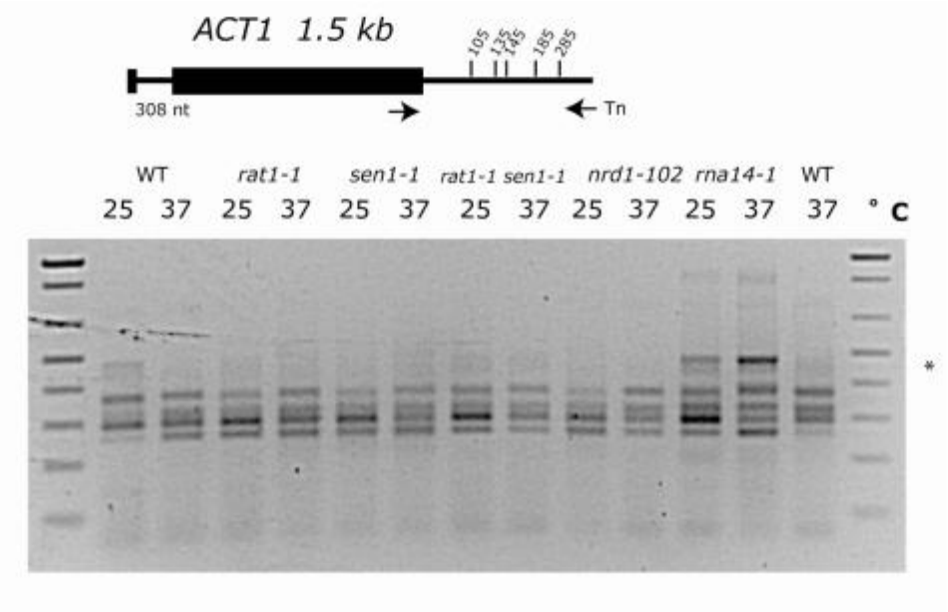

Figure S1. PolyA Site Selection Is Unaffected in *sen1-1* Cells, Related to Figure 1
3’ RACE analysis of polyadenylated RNAs (using indicated *ACT1* primer) produced from *ACT1* showing that polyA site selection is unaffected in *sen1-1* in contrast to *rna14-1* cells.

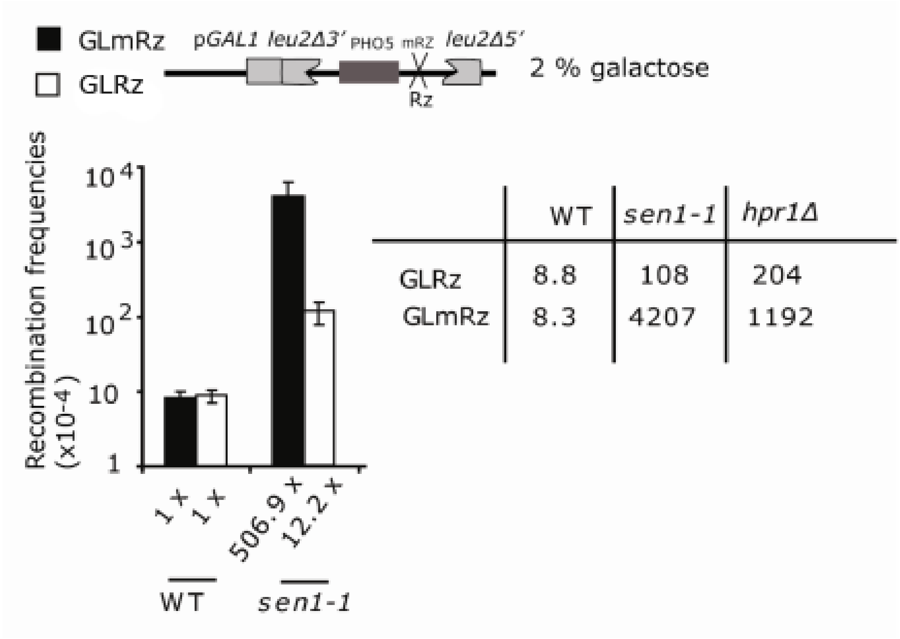

Figure S2. Affect of Transcript Cleavage on *sen1-1* Induced TAR, Related to Figure 2

Diagram of recombination substrates GLRz and GLmRz and the quantification of recombination events. WT and *sen1-1* cells were grown for 4 days at 30˚ C (selected for plasmid and supplemented with 2 % galactose), before recombinants were selected on leucine deficient plates.

	WT	WT +AID	*sen1-1*	*sen1-1* +AID
Mutations at C	1	1	1	12
Mutations at G	0	2	2	6
Mutations at WRC	0	0	1	12
Mutations at GYW	0	1	0	5
Point mutations	5	3	3	24
Total sequenced	14	10	47	57

Figure S3. Strand Specific Identification of Point Mutations on *URA3*, Related to Figure 3

The point mutations depicted in Figure 3C occurred in the AID specific sequence motifs; W = A or T, R = A or G, Y = C or T.

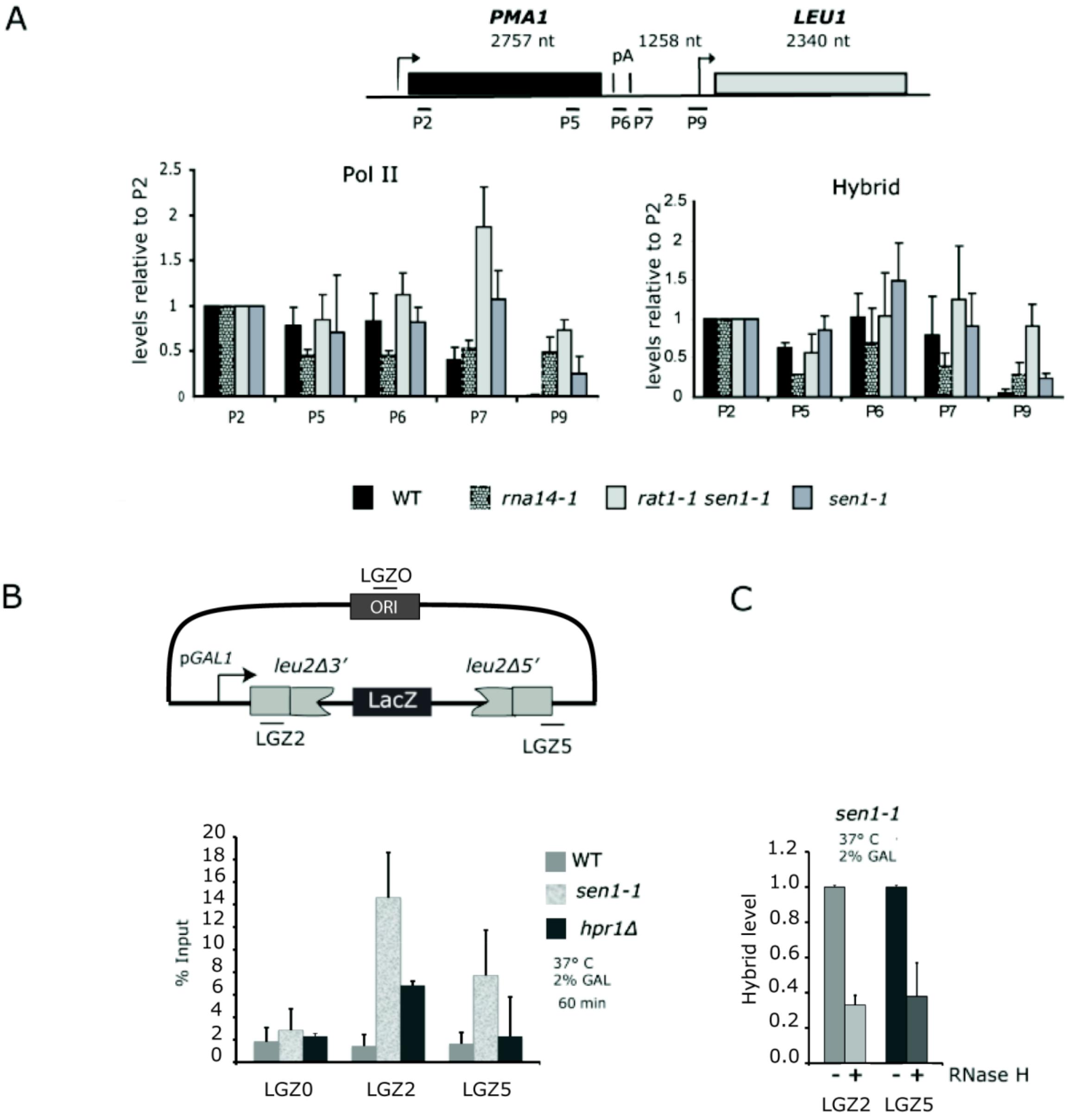

Figure S4. R-Loops Accumulate in *sen1-1* Cells, Related to Figure 4

A. ChIP analysis over *PMA1* with 8WG16 (Pol II) and S9.6 (RNA:DNA hybrid) antibodies. See diagram for primer positions. Chromatin was isolated from cells grown at 37˚ C for 150 min or 90 min (*rna14-1*).

B. DIP on pGLacZ showing lack of R-loop accumulation over non-transcribed ori region of plasmid. Growth conditions and DIP analysis were performed as described in Figure 4A and Experimental Procedures. Diagram indicates primer positions.

C. DIP signals originating from LGZ2 and LGZ5 are reduced by RNase H digestion. Prior to immunoprecipitation chromatin was incubated at 37˚ C for 1h in the presence or absence of RNase H.

A

transcripts:	CUTs	ORF-T	other	SUTs
all	925	5171	329	847
DE	218	381	57	157

B

DDCC \ DE	FALSE	TRUE
FALSE	4063	347
TRUE	727	34

C

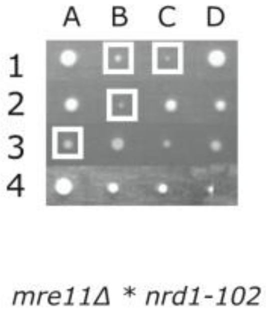

Figure S5. Transcriptome Analysis of *sen1-1* and Specificity of *sen1-1* Genetic Interaction to MRX, Related to Figure 5

A. Table to summarize differentially expressed (DE) genes in *sen1-1*. First row shows total numbers of transcripts by type in the *S. cerevisiae* genome (CUTs; ORF-Ts, ORF transcripts; SUTs, Stable Unannotated Transcripts as defined in Xu *et al.*(2009)). Second row shows those significantly differentially expressed of each class ($P < 0.01$). See Table SIV for complete data set

B. Identification of DE genes that have been classified to be involved in DNA damage and cell cycle progression (DDCC, as compiled in von Attikum *et al.* (2004) $P < 0.01$, Supplemental Methods). Thus, of 727 DDDC genes only 34 are DE. DDCC genes are therefore significantly depleted in DE genes (Fisher's test, $P < 2 \times 10^{-16}$).

C. Other mutants of NRD, such as *nrd1-102* show no synthetic interaction with MRX.

Table S1. Strains Used in This Study, Related to the Experimental Procedures

Strain	Genotype	Reference
AYW3-1B	Mat a *ade2-1 his3 ura3trp1 can1-100 leu2-k::ADE2-URA3::leu2-k*	(Santos-Rosa and Aguilera, 1995)
Dat1-1	Mat α *leu2Δ1 ura3-52 trp1 Δ63 rat1-1*	N.J. Cole
DUY1339	Mar α *leu2-3 ura3-52 pep4-3 rpb1-1 sen1-1*	(Ursic et al., 2004)
FD-4A	Mat *a leu2 ura3 rat1-1 sen1-1*	(Kawauchi et al., 2008)
FD-4B	Mat α *leu2 trp1Δ63 ura3 sen1-1*	(Kawauchi et al., 2008)
FD-4C	Mat *a leu2 ura3 rat1-1*	(Kawauchi et al., 2008)
FD-4D	Mat α *leu2 trp1Δ63 ura3*	(Kawauchi et al., 2008)
FWYI	Mat a *leu2-3,-112 ura3-52 pep4-3 trp1 sen1-1*	(Ursic et al., 1997)
U768-1C	Mat a *ade2-1 his3-11,15 leu2-3,112 trp1-1 ura3-1 hrp1Δ::HIS3 can1-100*	(Aguilera and Klein, 1990)
JDY-1C	Mat *a his3 leu2 met15Δ0 trp1Δ63 ura3 nrd1-102*	(Rondon et al., 2009)
KS2B	Mat a *ade3::GAL HO his3-11,15 leu2::SFA ura3-1 trp1-1 yKu70Δ::KAN sen1-1*	This study
KS4A	Mat α-inc *ade3::GAL HO his3-11,15 leu2::SFA ura3-1 trp1-1 yKu70Δ::KAN sen1-1*	This study
MF4C	Mat α *his3Δ leu2 ura3 trp1-1 mus81Δ::KAN sen1-1*	This study
MF5B	Mat α *his3Δ leu2 ura3 mus81Δ::KAN sen1-1*	This study
mft1Δ	Mat a *ade2-1 his3-11,15 leu2-3,112 trp1-1 ura3-1 can1-100 mfy1Δ::KAN*	(Gomez-Gonzalez and Aguilera, 2007)
Mre11-H125N	Mat a *ade2-1 his3-11,15 leu2-3,112 trp1-1 ura3-1 can1-100 mre11-H125N*	(Moreau et al., 1999)
MRS3A[a]	Mat α *his3-11,15 leu2-3,112 ura3 trp1-1*	This study
MRS3B[a]	Mat a *ade2-1 his3-11,15 leu2-3,112 ura3 trp1-1 sen1-1*	This study
MRS3C[a]	Mat a *his3-11,15 leu2-3,112 ura3 trp1-1*	This study
MRS3D[a]	Mat α *ade2-1 his3-11,15 leu3-3,112 ura3 trp1-1 sen1-1*	This study
RF1B	Mat α *his3-11,15 leu2-3,112 trp1-1 ura3-1 can1-100 ade3::GALHO rad52Δ::KAN sen1-1*	This study
RF3A	Mat a *his3-11,15 leu2-3,112 trp1-1 ura3-1 can1-100 ade3::GALHO rad52Δ::KAN sen1-1*	This study
RF7C	Mat a *his3-11,15 leu2-3,112 trp1-1 ura3-1 can1-100 (ade3::GALHO)? rad52Δ::KAN sen1-1*	This study
SLEU2K 9A	Mat a *his3 leu3 trp1-1 leu2K::ADE2-URA3::leu2k sen1-1*	This study
SS2A	Mat α *ade2-1 his3 lys2Δ leu2 trp1-1 ura3 sgs1Δ::KAN sen1-1*	This study

SS8B	Mat a *ade2-1 his3 leu2 ura3 sgs1Δ::KAN sen1-1*	This study
SSr1D	Mat α *his3Δ leu2 ura3 trp1-1 srs2Δ::KAN sen1-1*	This study
SSr2B	Mat a *his3Δ leu2 ura3 trp1-1 srs2Δ::KAN sen1-1*	This study
U674	Mat a-inc *ade2-1 ade3:: GAL-HO his3-11,15 leu2Δ::SFA trp1-1 ura3-52 rad50Δ::KAN*	(Malagon and Aguilera, 2001)
W303-1A	Mat a *ade2-1 his3-11,15 leu2-3,112 trp1-1 ura3-1 can1-100*	A. Aguilera
WF1A	Mata *his3-11,15 leu2-3,112 ura3-1 trp1-1*	This study
WF1B	Mat α *his3-11,15 leu2-3,112 ura3-1 trp1-1 sen1-1*	This study
Wn8b	Mat α *his3 leu2-112,3 ura3 trp1-1 nrd1-101*	This study
WRS52-4B	Mat a-inc *ade2 ade3:: GAL HO leu2Δ: SFA his3-11,15 trp1-1 ura3-52 rad50Δ::KAN*	(Gonzalez-Barrera et al., 2003)
WSKU	Mat α-inc *ade3::GAL HO his3-11,15 leu2::SFA ura3-1 trp1-1 yKu70::KAN*	Cortés-Ledesma & A. Aguilera in preparation.
WSM11	Mat a-inc *ade2-1 ade3::GAL HO his3-11,15 leu2::SFA trp1-1 ura3-1 mre11Δ::KAN*	(Gonzalez-Barrera et al., 2003)
Y01331	Mat α *his3Δ1 leu2Δ0 met15Δ0 lys2Δ0 ura3Δ0 srs2Δ::KAN*	Euroscarf
Y04222	Mat a *his3Δ1 leu2Δ0 met15Δ0 ly2sΔ0 ura3Δ0 mus81Δ::KAN*	Euroscarf
Y10775	Mat α *his3Δ1 leu2Δ0 met15Δ0 lys2Δ0 ura3Δ0 sgs1Δ::KAN*	Euroscarf
YPN103	Mat α *ade2 can1-100 his3-11,15 leu2-3,113 trp1-1 ura3-1 nab3-11*	(Conrad et al., 2000)
Δ3YBA 1B	Mat α *his3 leu3 leu2K::ADE2-URA3::leu2k*	This study

.

Table S2. Plasmids Used in This Study, Related to the Experimental Procedures

Plasmid	Backbone	Marker	Reference
pLAUR	pCM184	*TRP1*	(Jimeno et al., 2002)
P414GalAID	pRS414	*TRP1*	(Gomez-Gonzalez and Aguilera, 2007)
pLNA	pRS314	*TRP1*	(Prado et al., 1997)
pLNAT	pRS314	*TRP1*	(Prado et al., 1997)
pL	pRS316	*URA3*	A. Aguilera
pLYΔNS	pRS316	*URA3*	A. Aguilera
pLLacZ	pRS314/pSCH204	*TRP1*	(Chavez and Aguilera, 1997)
pLPHO5	pRS314	*TRP1*	(Prado et al., 1997)
pGLLacZ	pRS314	*TRP1*	(Piruat and Aguilera, 1998)
pGLPHO5	pRS314	*TRP1*	
P415GLG	pRS415GAL1	*LEU2*	(Gomez-Gonzalez and Aguilera, 2007)
pKGG	pRS426	*URA3*	(Morillon et al., 2003)
PN46 Sen1(323)	yPLac111	*LEU2*	(Nedea et al., 2008)
PN55 Sen1(1212)	yPLac111	*LEU2*	(Nedea et al., 2008)
PN4 Vector	yPLac111	*LEU2*	(Nedea et al., 2008)
pRad52-GFP	pWJ1344	*LEU2*	(Lisby *et al.*, 2001)
pYRNH201	BG1805	*URA3*	Openbiosystems
pYSen1	pYES2	*URA3*	This study
pYES2	pYES2	*URA3*	Invitrogen

Table S3. Primers Used in This Study, Related to the Experimental Procedures

Adapter	TTCGTATACCCGGGTACCAA
HIS 5’	GGCACTGCCATTTTACCAAG
KGG 3’	CAAGAAGGATAGTAAGCTGGCAAAGTC
KGG 5’	CATCCTATGGAACTGCCTCGG
LacZ 3067 3	AGGGCACGCTGATTGAAG
LacZ 959 5’	CGACGGATCCCCTTTTTG
LGZ 3 3'	GCCCTCCTCCTTGTCAATATTAATG
LGZ 5 5'	GTTCATAGGGTAGACGAAACTATATACGCAATC
LGZ2 3'	GCATCGATAGCAGCACCACC
LGZ2 5'	GTCTGCCCCTAAGAAGATCGTCG
LGZ0 5’	CCCTGCCGCTTACCGGATAC
LGZ0 3’	TAGTTACCGGATAAGGCGCAG
Muc1 F	CTAAGGAAAAGCCTACACCCCCAACC
Muc1 R	GGAGTAGTAGTCTTCTTAGTGCATGTCTTAGATGTGG
P2R	CTTCTTTCTTCTTTTCAAAACTTCATCGGAAG
P5F	GGCTCCTGGTCTATCTGCTATTATTGATGC
P5R	CAATCCATAGACCCAAGAAGATTTCCAAATG
P6F	GAGCCAACAAGAATAAGCCGCTTATTTCC
P6R	GAAAAAGTACCATCCAGAGAAACCAATTATATCAAATC
P7F	GAATCGTCTTTATTATGGTCAAGGCTTTACGTC
P7R	GAGTGTCTGTATGGGCGCATAAACGTAAG
P9F	GGCGAAATTTGCCCGGTTTTGTC
P9R	GAAAATTCAGCGGAAACAGCGTGATGAG
Phased oligodT adapter	TTCGTATACCCGGGTACCAATTTTTTTTTTTTTTTTTV
scR1 3’	CACAATGTGCGAGTAAATCCTG
scR1 5’	GGCTGTAATGGCTTTCTGGTG
SNR13 3’	GGTCAGATAAAAGTAAAAAAAGGTAGCTTGAG
SNR13 5’	GGAAGTTTTTTCCTTTTTATATGATGAATATG
Telomere VI 3’	CTCGTTAGGATCACGTTCGAATCC
Telomere VI 5’	GCGTAACAAAGCCATAATGCCTCC
URA 3’	CCACATCATCCACGGTTCTA

Supplemental References

Aguilera, A., and Klein, H.L. (1990). HPR1, a novel yeast gene that prevents intrachromosomal excision recombination, shows carboxy-terminal homology to the Saccharomyces cerevisiae TOP1 gene. Mol Cell Biol *10*, 1439-1451.

Benjamini, Y., and Hochberg, Y. (1995). Controlling the false discovery rate: a practical and powerful approach to multiple testing. Journal of the Royal Statistical Society Series_ B *57*, 289-300.

Chavez, S., and Aguilera, A. (1997). The yeast HPR1 gene has a functional role in transcriptional elongation that uncovers a novel source of genome instability. Genes Dev *11*, 3459-3470.

Conrad, N.K., Wilson, S.M., Steinmetz, E.J., Patturajan, M., Brow, D.A., Swanson, M.S., and Corden, J.L. (2000). A yeast heterogeneous nuclear ribonucleoprotein complex associated with RNA polymerase II. Genetics *154*, 557-571.

Gomez-Gonzalez, B., and Aguilera, A. (2007). Activation-induced cytidine deaminase action is strongly stimulated by mutations of the THO complex. Proc Natl Acad Sci U S A *104*, 8409-8414.

Gonzalez-Barrera, S., Cortes-Ledesma, F., Wellinger, R.E., and Aguilera, A. (2003). Equal sister chromatid exchange is a major mechanism of double-strand break repair in yeast. Mol Cell *11*, 1661-1671.

Huber, W., Toedling, J., and Steinmetz, L.M. (2006). Transcript mapping with high-density oligonucleotide tiling arrays. Bioinformatics *22*, 1963-1970.

Jimeno, S., Rondon, A.G., Luna, R., and Aguilera, A. (2002). The yeast THO complex and mRNA export factors link RNA metabolism with transcription and genome instability. EMBO J *21*, 3526-3535.

Kawauchi, J., Mischo, H., Braglia, P., Rondon, A., and Proudfoot, N.J. (2008). Budding yeast RNA polymerases I and II employ parallel mechanisms of transcriptional termination. Genes Dev *22*, 1082-1092.

Kessler, M.M., Zhao, J., and Moore, C.L. (1996). Purification of the Saccharomyces cerevisiae cleavage/polyadenylation factor I. Separation into two components that are required for both cleavage and polyadenylation of mRNA 3' ends. J Biol Chem *271*, 27167-27175.

Lisby, M., Rothstein, R., and Mortensen, U.H. (2001). Rad52 forms DNA repair and recombination centers during S phase. Proc Natl Acad Sci U S A *98*, 8276-8282.

Malagon, F., and Aguilera, A. (2001). Yeast spt6-140 mutation, affecting chromatin and transcription, preferentially increases recombination in which Rad51p-mediated strand exchange is dispensable. Genetics *158*, 597-611.

Mancera, E., Bourgon, R., Brozzi, A., Huber, W., and Steinmetz, L.M. (2008). High-resolution mapping of meiotic crossovers and non-crossovers in yeast. Nature *454*, 479-485.

Minvielle-Sebastia, L., Beyer, K., Krecic, A.M., Hector, R.E., Swanson, M.S., and Keller, W. (1998). Control of cleavage site selection during mRNA 3' end formation by a yeast hnRNP. EMBO J *17*, 7454-7468.

Moreau, S., Ferguson, J.R., and Symington, L.S. (1999). The nuclease activity of Mre11 is required for meiosis but not for mating type switching, end joining, or telomere maintenance. Mol Cell Biol *19*, 556-566.

Morillon, A., Karabetsou, N., O'Sullivan, J., Kent, N., Proudfoot, N., and Mellor, J. (2003). Isw1 chromatin remodeling ATPase coordinates transcription elongation and termination by RNA polymerase II. Cell *115*, 425-435.

Nedea, E., Nalbant, D., Xia, D., Theoharis, N.T., Suter, B., Richardson, C.J., Tatchell, K., Kislinger, T., Greenblatt, J.F., and Nagy, P.L. (2008). The Glc7 phosphatase subunit of the cleavage and polyadenylation factor is essential for transcription termination on snoRNA genes. Mol Cell *29*, 577-587.

Piruat, J.I., and Aguilera, A. (1998). A novel yeast gene, THO2, is involved in RNA pol II transcription and provides new evidence for transcriptional elongation-associated recombination. EMBO J *17*, 4859-4872.

Prado, F., Piruat, J.I., and Aguilera, A. (1997). Recombination between DNA repeats in yeast hpr1delta cells is linked to transcription elongation. EMBO J *16*, 2826-2835.

Rondon, A.G., Mischo, H.E., Kawauchi, J., and Proudfoot, N.J. (2009). Fail-safe transcriptional termination for protein-coding genes in S. cerevisiae. Mol Cell *36*, 88-98.

Santos-Rosa, H., and Aguilera, A. (1995). Isolation and genetic analysis of extragenic suppressors of the hyper-deletion phenotype of the Saccharomyces cerevisiae hpr1 delta mutation. Genetics *139*, 57-66.

Smyth, G.K. (2004). Linear models and empirical bayes methods for assessing differential expression in microarray experiments. Stat Appl Genet Mol Biol *3*, Article3.

Ursic, D., Chinchilla, K., Finkel, J.S., and Culbertson, M.R. (2004). Multiple protein/protein and protein/RNA interactions suggest roles for yeast DNA/RNA helicase Sen1p in transcription, transcription-coupled DNA repair and RNA processing. Nucleic Acids Res *32*, 2441-2452.

Ursic, D., Himmel, K.L., Gurley, K.A., Webb, F., and Culbertson, M.R. (1997). The yeast SEN1 gene is required for the processing of diverse RNA classes. Nucleic Acids Res *25*, 4778-4785.

van Attikum, H., Fritsch, O., Hohn, B., and Gasser, S.M. (2004). Recruitment of the INO80 complex by H2A phosphorylation links ATP-dependent chromatin remodeling with DNA double-strand break repair. Cell *119*, 777-788.

Xu, Z., Wei, W., Gagneur, J., Perocchi, F., Clauder-Munster, S., Camblong, J., Guffanti, E., Stutz, F., Huber, W., and Steinmetz, L.M. (2009). Bidirectional promoters generate pervasive transcription in yeast. Nature *457*, 1033-1037.

Zhao, J., Kessler, M., Helmling, S., O'Connor, J.P., and Moore, C. (1999). Pta1, a component of yeast CF II, is required for both cleavage and poly(A) addition of mRNA precursor. Mol Cell Biol *19*, 7733-7740.
